# Rabies and Distemper Outbreaks in Smallest Ethiopian Wolf Population

**DOI:** 10.3201/eid2312.170893

**Published:** 2017-12

**Authors:** Jorgelina Marino, Claudio Sillero-Zubiri, Asefa Deressa, Eric Bedin, Alemayehu Bitewa, Fekadu Lema, Gebeyehu Rskay, Ashley Banyard, Anthony R. Fooks

**Affiliations:** University of Oxford, Oxford, UK (J. Marino, C. Sillero-Zubiri, E. Bedin);; International Union for Conservation of Nature Species Survival Commission Canid Specialist Group, Oxford (C. Sillero-Zubiri);; Ethiopian Public Health Institute, Addis Ababa, Ethiopia (A. Deressa);; Environmental, Forest, Wildlife Development and Protection Authority, Bahir Dar, Ethiopia (A. Bitewa);; Ethiopian Wolf Conservation Programme, Bale Robe, Ethiopia (J. Marino);; Ethiopian Wolf Conservation Programme, Bahir Dar (F. Lema, G. Rskay);; Animal and Plant Health Agency, Weybridge, UK (A. Banyard, A.R. Fooks)

**Keywords:** rabies, canine distemper virus, mortality, Ethiopia, wolf, viruses, Ethiopian wolf, *Canis simensis*

## Abstract

Widespread deaths recently devastated the smallest known population of Ethiopian wolves. Of 7 carcasses found, all 3 tested were positive for rabies. Two wolves were subsequently vaccinated for rabies; 1 of these later died from canine distemper. Only 2 of a known population of 13 wolves survived.

Canine diseases pose a growing threat to wildlife species of conservation concern worldwide. Although extensive oral vaccinations have eliminated rabies virus (RABV) from wild carnivore populations in some developed countries (*1*), elsewhere, the challenges to controlling diseases in endangered wildlife are many and persistent. Massive outbreaks of rabies and, more recently, canine distemper have repeatedly decimated populations of Ethiopian wolves (*Canis simensis*) in the Bale Mountains of Ethiopia, where more than half of a global population of ≈500 wolves live (*2*,*3*). Extensive efforts to control RABV in the reservoir population of sympatric domestic dogs have proved insufficient. Therefore, reactive vaccination of Ethiopian wolves, carried out in response to an outbreak in wolves, has been the primary mechanism to curtail mortality in the affected wolf populations in the Bale Mountains (*4*).

The fragile status of the Bale population highlights the conservation value of the other remaining, much smaller, wolf populations scattered throughout the highlands of Ethiopia. Models predict these small populations to be particularly vulnerable to disease outbreaks (*5*); however, no outbreaks had been detected outside Bale, either because they went unnoticed, because in small populations outbreaks die out before causing a major epizootic event, or both. We report consecutive rabies and canine distemper outbreaks among Ethiopian wolves in Delanta, in the Wollo highlands (*6*).

This group of wolves is the smallest extant wolf population; 13 wolves in 3 family packs lived in the remaining 20 km^2^ of Afroalpine habitat in 2015. The first wolf carcass was detected in late June 2016; by early September, 7 deaths had been confirmed. RABV infection was identified as the cause of death in all 3 of the carcasses tested, as well as in samples from 1 domestic dog concurrently found dead within wolf habitat (Table). A vaccination intervention was initiated in September 2016, when only 3 wolves were known to be alive; 1 adult male (>2 years of age) and 1 subadult female (1–2 years of age) were trapped (*7*) and parenterally inoculated with Nobivac Rabies (Merck Animal Health, Madison, NJ, USA) (*4*). In December 2016, the female wolf was found dead and tested positive for canine distemper virus (CDV) (Table); CDV was also detected in a dog carcass found concurrently in the vicinity of the wolf range. In late May, the vaccinated male was still alive and was observed until at least April 2017 with an unknown adult female.

**Table:** Characteristics of and test results for Ethiopian wolf and domestic dog carcasses recovered in Delanta, Ethiopia, 2016*

Date found	Species	Age and sex	Postmortem	Tested for RABV†	Tested for CDV‡
Jun 26	Ethiopian wolf	Juvenile female	No	NA	NA
Jun 28	Ethiopian wolf	Juvenile male	Yes	No	No
Jul 07	Ethiopian wolf	Adult male	Yes	Positive	No
Jul 11	Ethiopian wolf	Juvenile female	Yes	Positive	No
Jul 18	Ethiopian wolf	Juvenile female	No	NA	NA
Aug 12	Ethiopian wolf	Adult male	No	NA	NA
Sep 01	Domestic dog	Adult male	Yes	Positive	Negative
Sep 07	Ethiopian wolf	Adult female	Yes	Positive	Negative
Nov 27	Domestic dog	Adult male	Yes	Negative	Positive
Dec 27	Ethiopian wolf	Adult female	Yes	Negative	Positive
*CDV, canine distemper virus; NA, not applicable; RABV, rabies virus. †Rabies diagnostic reverse transcription PCR was performed as described previously (*6*). ‡CDV diagnostic reverse transcription PCR as performed as described previously (*3*).					

Evidence indicates a first outbreak of rabies, overlapping or followed soon after by a canine distemper outbreak. Confirmation of disease in contemporarily recovered dog carcasses is consistent with a pattern of transmission from reservoir domestic dogs to their wild relatives (as observed in the Bale Mountains [*8*]), with disastrous consequences for the small Delanta population, which harbored <20 wolves before the epizootic events. Although the larger Bale wolf population has recovered from epizootic events in the past (*2**,**9*), smaller populations are expected to be less resilient, a factor exacerbated by their virtual isolation from other wolf populations. Modeling has predicted a high extinction risk if Ethiopian wolf populations are affected by consecutive epizootic events over a short period of time (*5*). The combined exacerbated effects of RABV and CDV infection were first described in 2010 in the Bale Mountains (*3*).

Although the loss of Afroalpine habitats is bound to determine the fate of Ethiopian wolf populations (2 extinctions were recorded in areas of a similar size to that of Delaware during 1999 and 2010) (*2*), incursions of infectious diseases can drive local extinctions. Preemptive vaccination, in combination with actions to protect the habitat of this specialized predator, could greatly reduce the risk of populations becoming extinct, even if a relatively low proportion of the wolves is successfully vaccinated (*4*). Recently, SAG2, an oral rabies vaccine, was successfully tested in Ethiopian wolves (*10*), and a CDV parenteral vaccination trial is ongoing, with positive preliminary results. We propose proactive vaccination of Ethiopian wolves across their distribution as an effective and urgently needed strategy to protect the species from extinction. This program should be part of an integrated disease control plan that also includes controlling disease in domestic dogs, limiting contact between dogs and wolves, and conducting policy and education interventions to reduce the size and roaming behavior of local dog populations (*2*).
